# Supplementary material for: Enhanced Anti-Nociception by Novel Dual Antagonists for 5-HT2AR and mGluR5 in Preclinical Models of Pain
Source: Biomolecules. 2025 Oct 15;15(10):1456. doi: 10.3390/biom15101456 (PMC12564658; doi:10.3390/biom15101456)
Supplement: Supplementary file 1 [file biomolecules-15-01456-s001.zip › biomolecules-3887795-supplementary.pdf]

Supplementary figures

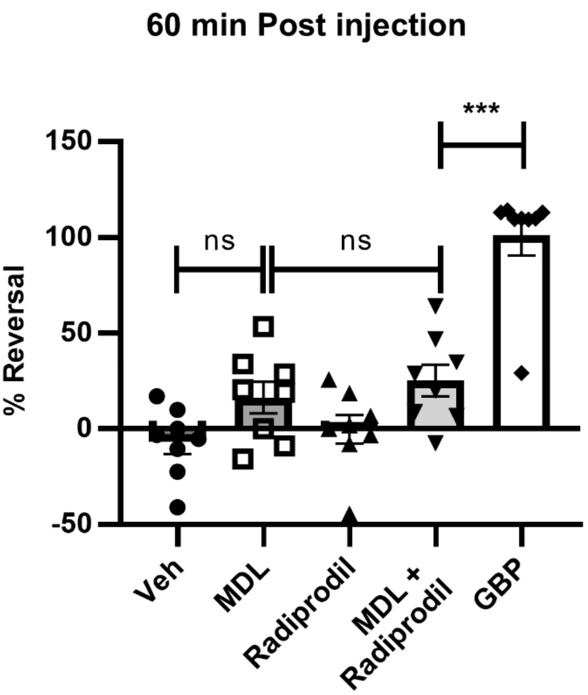

Figure S1. No efficacy enhancement of dual antagonism against 5-HT2AR and NMDA receptor in SNL model. Percent (%) reversal of the paw withdrawal threshold in the SNL model after treatment of vehicle (2:8 DMA/PG, s.c.), MDL (5 mg/kg, s.c.), Radiprodil (30 mg/kg, s.c.), a combination of MDL and Radiprodil, or GBP (65 mg/kg, i.p.) (N = 8 rats per group). Error bars present S.E.M., "ns" means no significance ($P > 0.05$); $^{***}P < 0.001$, One-way ANOVA.

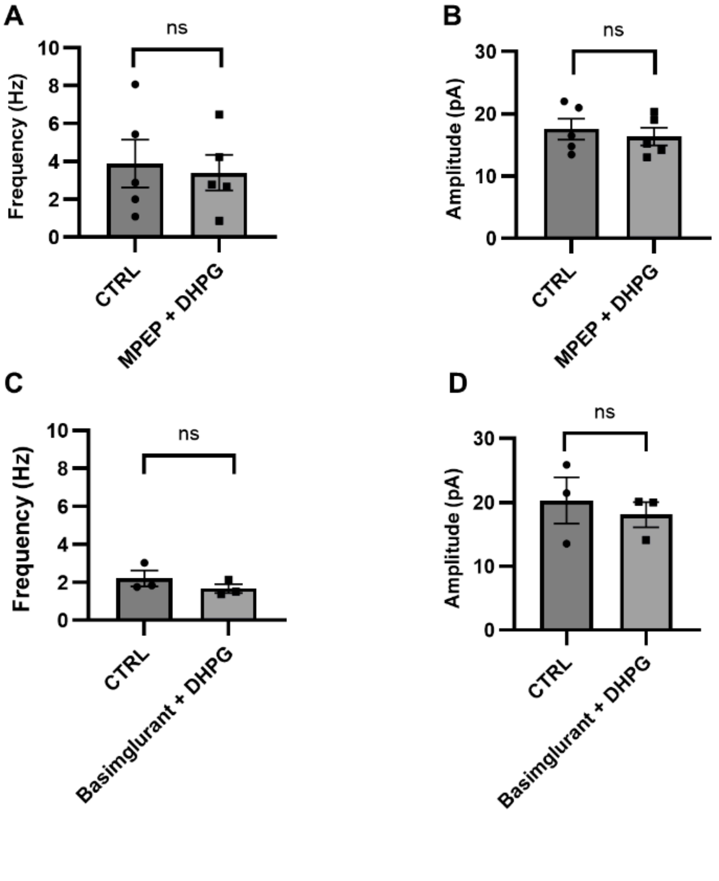

Figure S2. DHPG-induced sEPSC in the presence of MPEP and Basimglurant. **(A)** Frequency (Hz) changes in the presence of MPEP (10 μM) and MPEP plus DHPG (3 μM), Control (CTRL): 3.9 ± 1.1; MPEP+DHPG: 3.4 ± 0.8, n=5 / N=1). **(B)** Amplitude (pA) changes in the presence of MPEP and MPEP plus DHPG (3 μM) (CTRL: 17.6 ± 1.5; DHPG + MPEP: 16.3 ± 1.3, n=5 / N=1). **(C)** Frequency changes in the presence of Basimglurant (10 μM) and Basimglurant plus DHPG (3 μM) (CTRL: 2.2 ± 0.3; DHPG + Basimglurant: 1.7 ± 0.2, n=3 / N=1). **(D)** Amplitude changes in the presence of Basimglurant (10 μM) and Basimglurant plus DHPG (3 μM) (CTRL: 20.3 ± 2.9; DHPG + Basimglurant: 18.1 ± 1.6, n=3 / N=1). Error bars present S.E.M., "ns" means no significance ($P > 0.05$); *$P < 0.05$; **$P < 0.01$; ***$P < 0.001$, Two-tailed t-test.

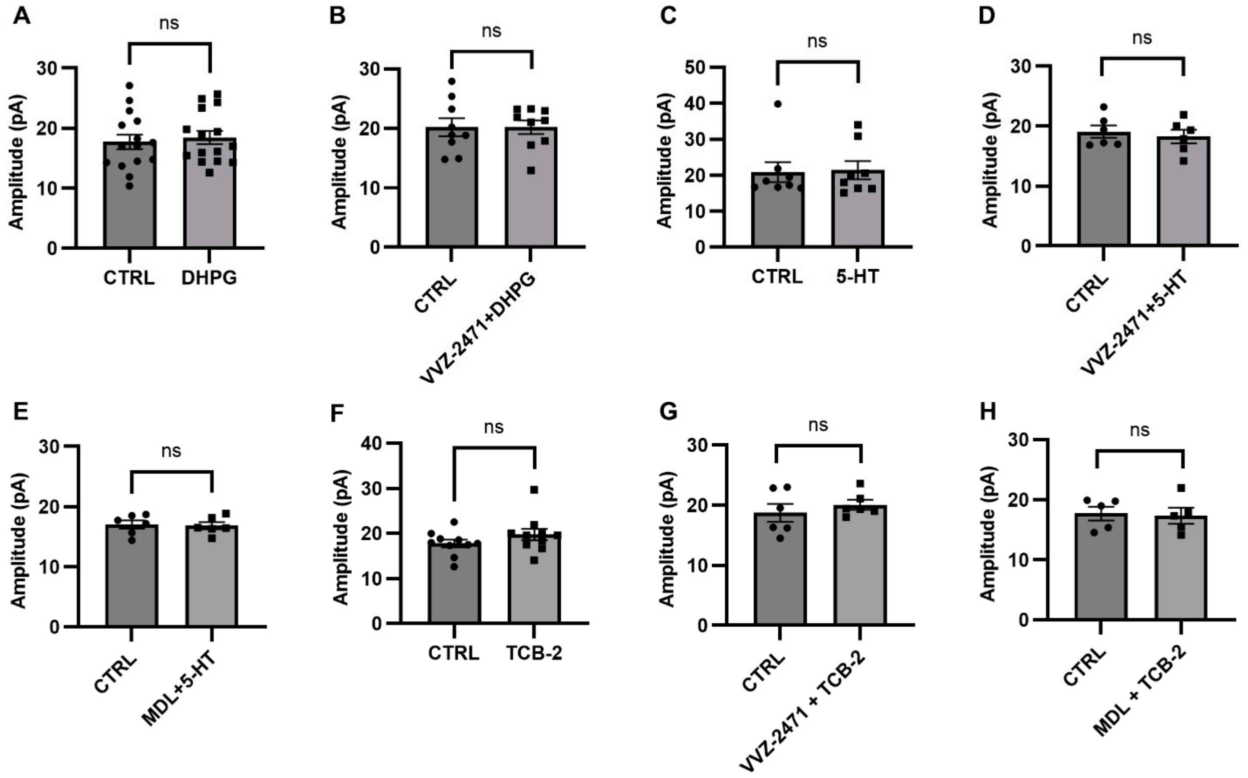

Figure S3. No significant effects of VVZ-2471 on the amplitudes of sEPSC. **(A)** Amplitude (pA) changes by DHPG alone (3 μM, Control (CTRL): 17.7 ± 1.2; DHPG: 18.5 ± 1.1, n=15 / N=9). **(B)** Amplitude changes by DHPG in the presence of VVZ-2471 (10 μM) (CTRL: 20.2 ± 1.4; DHPG + VVZ-2471: 20.1 ± 1.1, n=9 / N=3). **(C)** Amplitude changes by 5-HT (50 μM, CTRL: 20.8 ± 2.6; 5-HT: 21.4 ± 2.3, n=8 / N=6). **(D)** Amplitude changes by 5-HT in the presence of VVZ-2471 (CTRL: 19.1 ± 1.0; 5-HT + VVZ-2471: 18.2 ± 1.0, n=6 / N=3). **(E)** Amplitude changes by 5-HT in the presence of MDL11,939 (MDL) (10 μM, CTRL: 17.0 ± 0.6; 5-HT + MDL: 16.8 ± 0.5, n=6 / N=4). **(F)** Amplitude changes by TCB-2 alone (100 μM, CTRL: 17.7 ± 0.8; TCB-2: 19.7 ± 1.2, n=10 / N=5). **(G)** Amplitude changes induced by TCB-2 in the presence of VVZ-2471 (10 μM, CTRL: 18.7 ± 1.3; TCB-2 + VVZ-2471: 20.1 ± 0.7, n=6 / N=3). **(H)** Amplitude changes induced by TCB-2 in the presence of MDL (10 μM, CTRL: 17.5 ± 1.2; TCB-2 + MDL11,939: 17.4 ± 1.3, n=5 / N=3). Error bars present S.E.M., "ns" means no significance ($P > 0.05$), Two-tailed t-test.

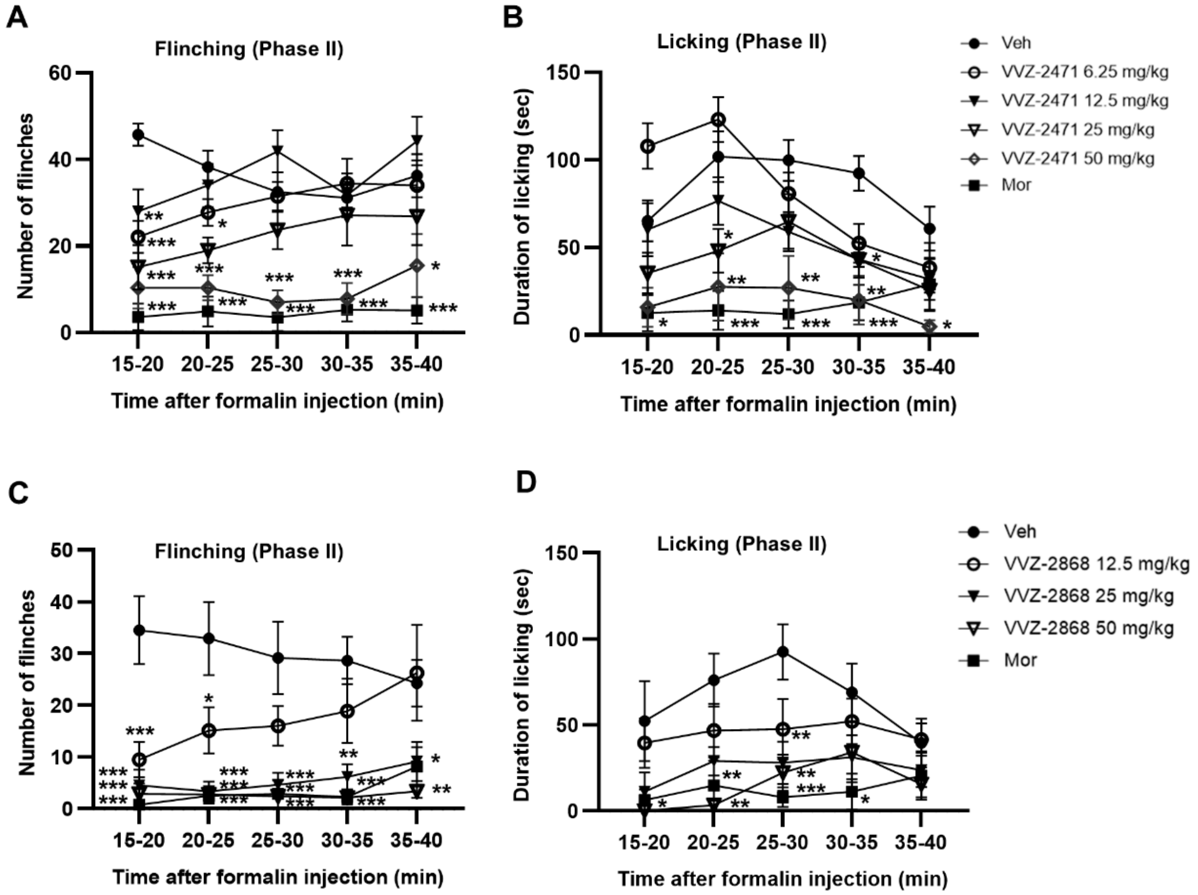

Figure S4. Anti-nociceptive effects of dual antagonists against 5-HT2AR and mGluR5 in the formalin-induced pain model. Time-course of flinches **(A, C)** and licking **(B, D)** responses induced by formalin injection to the rats treated with VVZ-2471 (p.o.), VVZ-2868 (p.o.), or Morphine (MOR, 2 mg/kg, s.c.). N = 8 rats per group. Error bars present S.E.M., *$P < 0.05$; **$P < 0.01$; ***$P < 0.001$ *vs* Vehicle, Two-way ANOVA.

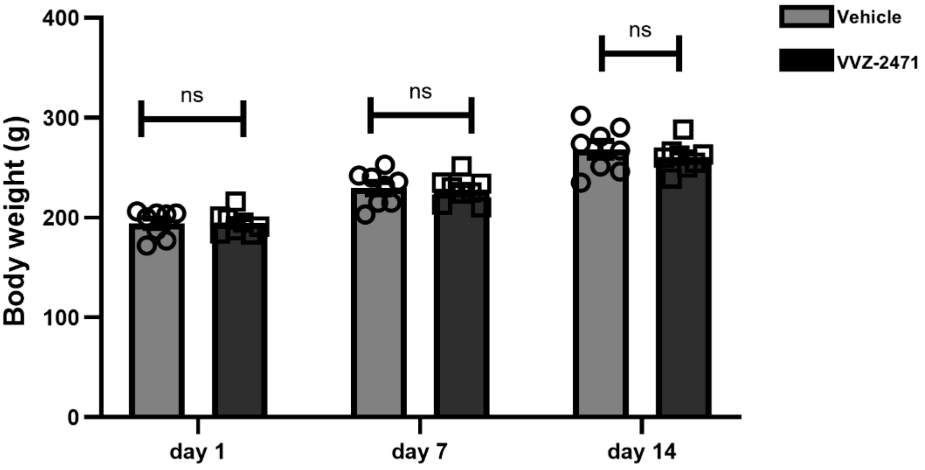

Figure S5. Effect of VVZ-2471 on body weight. VVZ-2471 (25 mg/kg, p.o.) and vehicle (p.o.) were administered once a day for 14 consecutive days (N=8 rats per group). Body weight measured on Days 1, 7, and 14. Error bars present S.E.M., “ns” means no significance ($P > 0.05$), One-way ANOVA.
